# Standardized assay for assessment of minimal residual disease in blood, bone marrow and apheresis from patients with plasma cell myeloma

**DOI:** 10.1038/s41598-019-39631-2

**Published:** 2019-02-27

**Authors:** Agnieszka Blum, Katy Haussmann, Mathias Streitz, Stephan Schlickeiser, Carola Tietze-Buerger, Igor Wolfgang Blau, Lutz Uharek

**Affiliations:** 1 Charité Stem Cell Facility, Charité – Universitätsmedizin Berlin, corporate member of Freie Universität Berlin, Humboldt-Universität zu Berlin, and Berlin Institute of Health, Augustenburger Platz 1, Berlin, 13353 Germany; 2 Department of Haematology, Oncology, Tumor Immunology, Charité – Universitätsmedizin Berlin, corporate member of Freie Universität Berlin, Humboldt-Universität zu Berlin, and Berlin Institute of Health, Augustenburger Platz 1, Berlin, 13353 Germany; 3 Institute of Medical Immunology, Charité – Universitätsmedizin Berlin, corporate member of Freie Universität Berlin, Humboldt-Universität zu Berlin, and Berlin Institute of Health, Augustenburger Platz 1, Berlin, 13353 Germany

## Abstract

The recent advances in myeloma treatment result in significantly better outcomes, defined as increased progression free survival (PFS) and overall survival (OS). Since there is a proven correlation between the extend of response and prolonged survival, there is an urgent need for highly sensitive assays for the detection of minimal residual disease (MRD). Next generation flow cytometry has become a valuable approach for sensitive evaluation of the depth of complete response (CR). Here, we report the diagnostic performance and validation results of a single-tube 9-color panel assay. The validation design included intra-assay analysis measuring accuracy, inter-assay analysis estimating method’s linearity and precision and inter-assay analysis evaluating repeatability. Furthermore, in inter-operator analysis assessed the comparability of the result analysis of different operators. Staining stability was evaluated in age-of-stain experiments. Our validation results show that a reliable detection of residual myeloma cells is feasible to a detection level of 10^−5^ with a single-tube assay for a variety of materials (peripheral blood, bone marrow and stem cell apheresis). This study establishes highly sensitive, fully standardized approach for MRD detection in myeloma that is ready for implementation in routine diagnostic laboratories.

## Introduction

Plasma cell myeloma is a hematologic neoplasm characterized by the proliferation of malignant plasma clones. With targeted therapies available, a considerable number of patients can achieve complete response and have a significantly better outcome, defined as increased progression free survival and overall survival^1,2^. However, only 3 to 10% of plasma cell myeloma patients who have received high dose therapy will remain in complete remission for more than ten years^3^, while the majority will eventually relapse and undergo further treatment. Since there is a correlation between the extend of response and prolonged survival, there is an urgent need for highly sensitive assays for the detection of minimal residual disease (MRD)^4,5^. MRD is a more sensitive measure of response than conventional criteria and was shown to have an enhanced predictive value in comparison to standard methods^5^. Thus, MRD detection is very important for deciding whether a patient will undergo relapse-appropriate treatment^2,6^.

Multiparameter flow cytometry enables robust and cost effective monitoring of minimal residual disease^7^ in plasma cell myeloma patients. Because of the increased number of simultaneously used fluorochromes huge variety of cells and subtypes with different characteristics can be assessed. This enables estimation of the MRD by detection and differentiation between normal and abnormal plasma cells.

In order for MRD assays to be highly specific and sensitive, a combination of immunophenotypic markers that are able to identify and discriminate between normal and abnormal plasma cells is required^1,8–10^. CD38 and CD138 were used as gating markers, while CD19, CD27, CD45, CD56, CD81, CD200 and CD117 allowed for the identification of the most frequent deviation from the normal plasma cell phenotype. In addition, the presence of CD45 allowed for further phenotypic characterization of plasma cells and their quantification relative to the leukocyte count.

In order to obtain a quantification limit (LOQ)^11^, defined as the lowest concentration at which the analyte can be quantified, in the magnitude of ≤10^−5^ (i.e. one abnormal plasma cell detected in a population of ≥100,000 leucocytes) the sample has to be enriched to a total leucocyte count of 3–5 million in a small volume (e.g. 100 μl) following blood cell counting. The obtained cell suspension has to be stained according to a standard operating procedure (SOP)^11,12^.

In this study, we present a highly sensitive and standardized procedure for assessing minimal residual disease in patients with plasma cell myeloma in peripheral blood, bone marrow as well as in apheresis product. Our results show that our assay due to its highly discriminative combination of antibodies and effective gating strategy can be easily applied and validated in high throughput flow cytometry laboratories.

## Materials and Methods

### Qualification of instruments and good manufacturing practice (GMP) training

Qualification of all cytometers used in the study was preceded by risk analysis using the Ishikawa (fishbone diagram) and risk mitigation strategy performed according to failure modes and effects analysis (FMEA)^13^. Moreover, all cytometers underwent qualification based on written SOPs. All procedures were described in SOPs and the technical staff was adequately trained in using the SOP Guard Software.

### Blood and apheresis specimen collection

The study was approved by the Ethics Committee of the Charité – Universitätsmedizin, Berlin, Germany. All experiments were performed in accordance with relevant guidelines and regulations. Healthy individuals and plasma cell myeloma patients undergoing stem cell apheresis at the Charité – Universitätsmedizin, Berlin, Germany were recruited for this study. Written informed consent was obtained from all participants. Blood was collected into vacutainers (BD, Heidelberg, Germany) containing EDTA for anticoagulation. Apheresis samples were collected with the Spectra Optia® Apheresis System (Terumo BCT) using the Continuous Mononuclear Cell Collection (CMNC) protocol.

### Myeloma cell line

For the inter-assay analysis the myeloma cell line U266 was used. This cell line was established from the peripheral blood of a 53-year-old male with IgE-secreting myeloma in 1968. U266 myeloma cells express the following markers: CD38weak+, cyCD79a+, CD138+, HLA-DR+, cylambda+, while they are negative for CD3−, CD5−, CD10−, CD13−, CD19−, CD20−, CD34−, CD37−, sm/cyIgG−, sm/cyIgM−, sm/cykappa−, smlambda−.

### Materials

Premixed, dry reagent cocktail (DuraClone RE PC antibody panel) as well as CD117 ECD (Clone: 104D2D1) and CD3 ECD (Clone: UCHT1) antibodies used for the assessment of residual abnormal plasma cells and T cells were obtained from Beckman Coulter (Marseille, France). DuraClone RE PC tubes contained dried antibodies dosed to stain 3–5 × 10^6^ leukocytes in 100–200 μl volume: CD81 FITC (Clone: JS64)^14^; CD27 PE (Clone: 1A4CD27)^15^; CD19 PC5.5 (Clone: J3–119)^16^; CD200 PC7 (Clone: OX-104)^17^; CD138 APC (Clone: B-A38)^18^; CD56 APC-A750 (Clone: N901)^19^; CD38 Pacific Blue (Clone: LS198-4-3)^20^; CD45 Krome Orange (Clone: J.33).

### Sample staining procedure

Flow cytometric analysis was performed in the same single laboratory (Charité, Stem Cell Facility, Berlin, Germany) according to the manufacturer’s instructions (Beckman Coulter (Marseille, France) and according to the principles outlined by the European Myeloma Network^21^ (Tables 1, 2).

**Table 1:** Overview of the validation process.

Observed variability	Intra-assay	Verification of drop-in options	Inter-assay	Inter-assay	Inter-operator	Age of stain	Background from blank
Performance Characteristic	Accuracy	Accuracy	Precision/Linearity	Repeatability	Variability of data analysis results	Stability of prepared samples	Limit of blank (LOB)
Sample type	normal whole blood spiked with patient apheresis product	5 × apheresis products 3 × bone marrow Drop-in variants: CD3/CD117 ECD	normal whole blood spiked with U266 cells	1 × peripheral blood, 1 × bone marrow, 1 × apheresis product	list mode data obtained from patient apheresis or whole blood	1 × bone marrow (0,4,6,8 h), 1 × patient apheresis product (0, 4, 6, 8, 18 h), 1 × peripheral blood (0, 4, 6, 8, 24 h)	25 × normal whole blood
No. of samples with different plasma cell concentrations	6 (serial dilution)	8 (individual samples)	6 (serial dilution)	1 (for each type of material)	25 (individual samples)	1 (for each type of material, 4–5 different time points)	1
No. of replicates	3	1	3 in 5 different normal donors	10	1	10	1 in 25 different normal donors
Total no. of samples	18	8	90	30	25 (×5 different data analysis operators)	140 (including the repeatability samples)	25

**Table 2:** Comparison of different flow cytometry based MRD methods.

		DURAClone RE PC	MKSSC^32^	Euroflow
Assay format	No of tubes/no of colours/	1/8/	1/10/	2/6/
	Minimal cytometer configuration	blue/4-red/2-violet/2	blue/4-red/3-violet/3	blue/4-red/2-violet/2
	Formulation	glassified layer in FACS tube, 1-test-unitized	liquid single-color antibodies	3 different lyophilized bulk mixes for reconstitution
	Compensation approach	8 colors, dry single-color antibodies, 1-test-unitized	liquid single-color antibodies	4 color liquid single color antibodies (tandem dyes only), each tube with different compensation for dark red channel on red laser
	Storage & shipping	room temperature	cold storage	cold storage
	Marker selection according to consensus^21^	CD200 added, CD117 missing (can be added as drop in)	kappa, lambda added	kappa, lambda added CD27 and CD138 missing (can be added as drop in)
Mode of application	Sample type	bone marrow*, peripheral blood, apheresis product	bone marrow	bone marrow*
	Lysing method	bulk lysis of a volume equivalent to at least 5 × 10^6^ leukocytes	bulk lysis of a volume equivalent to 20 × 10^6^ leukocytes	bulk lysis of a volume equivalent to at least 20 × 10^6^ leukocytes (2 × 10 × 10^6^)
	Staining capacity	5 × 10^6^ leukocytes*	all recovered cells from bulk lysis	10 × 10^6^ leukocytes*
	Staining mode	surface	surface and intracellular	1 × surface; 1 × surface and intracellular
	Theoretical LOD/LOQ (20/50 positive events; %CD45+) based on 1/2 staining capacity	8 × 10^6^/2 × 10^5^	2 × 10^6^/5 × 10^6^ (10 × 10^6^ cells staining capacity)	4 × 10^6^/1 × 10^5^

Briefly, the number of leucocytes in the specimens was determined using the hematology analyzer (Sysmex IP300 (XP-350), Sysmex). Leucocytes were then lysed using Versafix solution (VersaLyse, Beckman Coulter supplemented with 0.25% IOTest Fixative Solution) at room temperature. Cell suspensions containing 3–5 × 10^6^ leukocytes were then centrifuged at 300 x g and resuspended in 100 μl phosphate buffered saline (PBS, Gibco). Cell suspensions were then transferred to DuraClone RE PC tubes and an appropriate volume of CD117 ECD antibody was added. After 15 min incubation at room temperature, cells were washed with PBS and centrifuged at 150 × g. The final pellet was resuspended in 500 μL PBS containing fixative solution (10% IOTest3 fixative solution, Beckman Coulter).

### Data acquisition

Sample acquisition was performed on 10-color, 3-laser NAVIOS flow cytometers (Beckman Coulter) using predefined settings. Debris was excluded by appropriate adjustment of the FSC recording trigger. Each sample was run twice in order to enhance the number of recorded events. Those duplicate readings were merged using the Kaluza analysis software prior to the final analysis.

Acquisition settings were defined according to the manufacturer’s instructions using the eight DuraClone RE PC compensation tubes as well as single CD117 or CD3 staining. Obtained photomultiplier tube (PMT) voltages were used to define target channels for all scatter and fluorescence detectors using calibration bead particles (Flow-Set Pro beads, Beckman Coulter). Matching of target channels was verified daily with a new calibration run to prevent target mismatch. Furthermore, all instruments underwent daily verification of optical alignment and fluidics using other calibration bead particles (Flow Check beads, Beckman Coulter).

### Data analysis

All acquired data files were analyzed using the Kaluza software, version 1.3 (Beckman Coulter). The two data files of the each stained sample were merged and analyzed. Cell doublets were excluded using either selection of events with highest FSC peak signals or with smallest width of FSC signal. Furthermore, cell debris was excluded from the analysis by using forward scatter time versus forward scatter dot plot. Dye aggregates were excluded from the analysis as outliers with high fluorescence intensities on the FITC detector and/or the near-infrared APC-AF750 detector. Absolute counts of the subpopulations were calculated in all panels by correlating CD45+ events with the white blood count obtained from all samples. Plasma cells were identified as events with high CD138 and high CD38 expression density. Abnormal phenotypes varied amongst patients and were identified by a combination of the following features: diminished expression of CD19, CD27, CD38, CD45, and/or CD81, overexpression of CD56, asynchronous expression of CD117 and CD200. Clusters of normal and abnormal plasma cells were identified using a 2D projection of all 9 fluorescent parameters (radar plot, Kaluza Analysis Software).

For the analysis of the inter-assay test, intra-assay test and inter-operator analysis, template analysis protocols were created for each set of experiments. Data files of the same sample were analyzed by loading the data files of each time point (inter-assay test) or parallel staining (intra-assay test) into the appropriate template. Accordingly, for the inter-operator analysis data, files were analyzed by 5 different operators using the same templates. Furthermore, each analysis included verification of compensation and, if applicable, minor adjustments. In case of age of stain assays analysis (for the 8-, 18- and 24 h specimens) the sideward scatter (SSC) parameter was adjusted when necessary.

Gated event counts were exported to Excel (Microsoft, Redmond, WA, USA) for the calculation of the frequencies of the subpopulations together with the respective mean fluorescence intensities and related standard deviation. Coefficients of variation (CVs) were calculated for each subpopulation frequency from replicates prepared from the same sample. The CVs obtained in intra- and inter-assay analysis were compared with the CVs as expected for the Poisson distribution characteristics.

### Design of validation approach

Design of the validation of the residual plasma cells measurement was based on the current recommendation^10,22,23^. Specifically, the validation design included intra-assay analysis measuring accuracy, inter-assay analysis estimating method’s linearity and precision and inter-assay analysis evaluating repeatability (Table 1). Furthermore, in inter-operator analysis the comparability of the result analysis of different operators was assessed. Stability of the staining was evaluated in age-of-stain experiments. For the intra-assay test, blood samples from healthy individuals were spiked with different concentrations of stem cell apheresis product from patients with plasma cell myeloma. For the inter-assay analysis blood from five healthy donors was collected and spiked with U266 cells at five different concentrations: 0,5000%; 0,0500%; 0,0050%; 0,0010%; 0,0005%; 0,0003%.

For the inter-assay analysis, whole blood, stem cell apheresis and bone marrow samples were taken from three patients with multiple myeloma. For the inter-operator analysis, a data set consisting of 25 files (22 unique files, 3 file doublets) was analyzed independently by five independent, trained operators.

## Results

### Intra-assay analysis: Accuracy

The aim of the intra-assay analysis was to evaluate the accuracy of assessment of minimal residual disease in patients with plasma cell myeloma. 18 samples from normal donors with 6 different concentrations of spiked U266 cells were assayed, recording 1,799 × 10^3^ ± 481 × 10^3^ CD45+ events. Samples were created by spiking whole blood from a healthy donor with patient apheresis product of known plasma cell frequencies as assayed by flow cytometry analysis. Final target frequencies ranged from 0.008–0.0005% of CD45+ anomalous plasma cells, representing 8 anomalous plasma cells in 1 × 10^5^ CD45+ to 1 anomalous plasma cell in 2 × 10^5^ CD45+ cells, respectively. Table 3 shows the results of normal samples spiked with patient apheresis products. CVs were in the expected range (Poisson noise) for all tested samples. Recovery of spiked cells was found at an average of 85% when referring to samples above the theoretical lower limit of quantitation (LLOQ) from 0.008% to 0.004% and 75% for samples above the theoretical lower limit of detection (LLOD) from 0.008% down to 0.002%. The theoretical lower limit of detection was calculated as the percentage of minimum 20 positive events in the total number of leucocytes acquired. Furthermore, the theoretical limit of quantification was calculated as the percentage of minimum 50 positive events in the total number of leucocytes acquired^11,24,25^.

**Table 3:** Results from validation runs on intra-assay variation/accuracy (^*^represents values below theoretical LLOQ; **represents spiked values below the theoretical LLOD).

Spiked %CD45	Recovered %CD45 (mean from 3 replicates)	Relative recovery of target frequency	Number of CD45+ (x10^3^, mean from 3 replicates)	Number of recovered events (mean from 3 replicates)	CV	mean^−1/2^ (“poisson noise”)	theoretical LLOD (20 cells min)	theoretical LLOQ (50 cells min)
0.0080%	0.0086%	107%	2,674	228	7%	7%	0.00075%	0.00187%
0.0060%	0.0048%	80%	2,037	98	8%	10%	0.00098%	0.00245%
0.0040%	0.0027%	68%	1,708	47	6%	15%	0.00117%	0.00293%
0.0020%*	0.0013%	64%	1,513	19	24%	23%	0.00132%	0.00330%
0.0010%**	0.0007%	69%	1,453	10	44%	32%	0.00138%	0.00344%
0.0005%**	0.0003%	57%	1,408	4	25%	50%	0.00142%	0.00355%

### Verification of drop-in options: Accuracy

**Figure 1:**
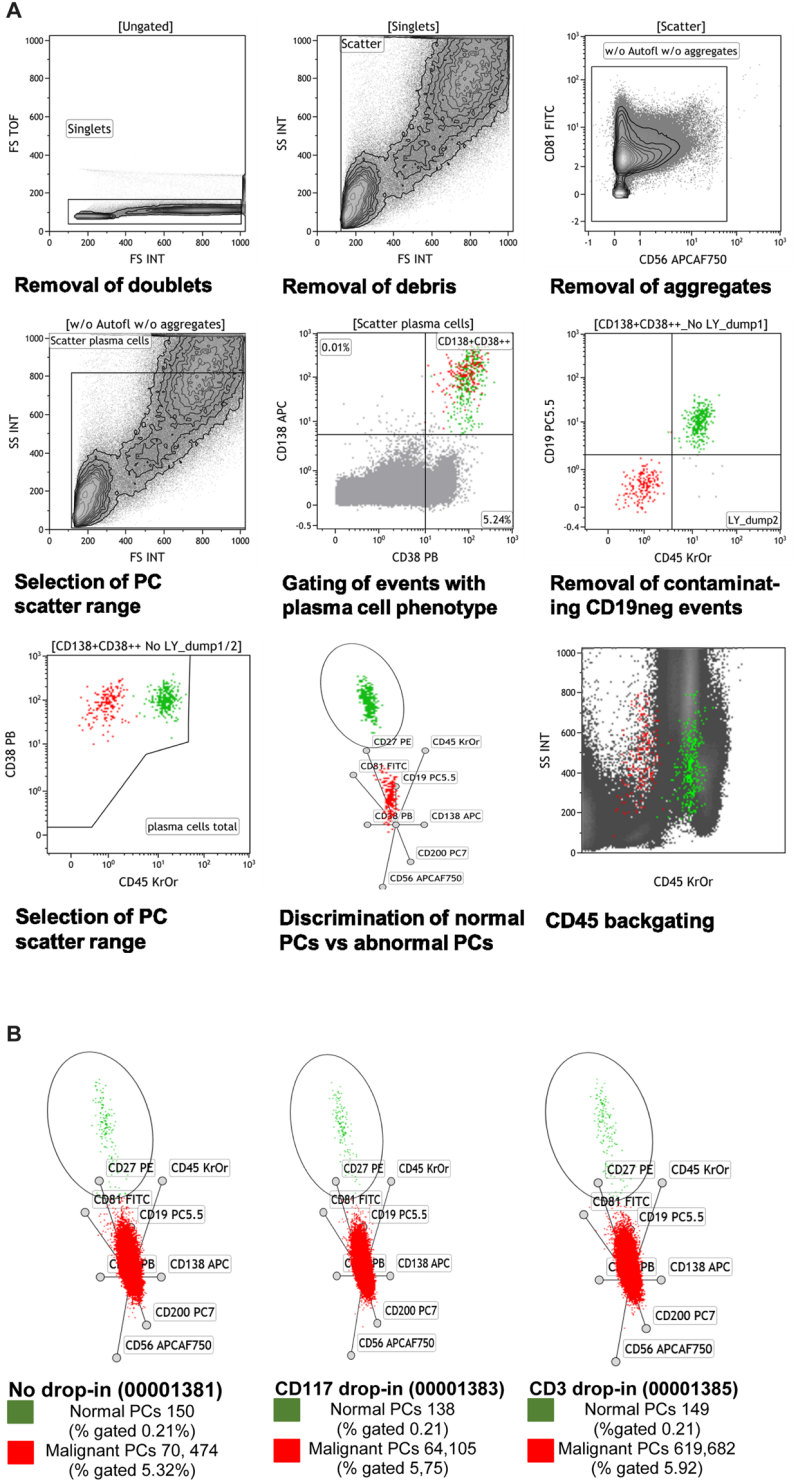
(**A)** Overview of the gating strategy for rare events plasma cells using the apheresis sample of a patient with plasma cell myeloma. Two compiled data files of lysed and stained samples were analyzed as follows: exclusion of non-single events, debris and dye aggregates. Furthermore, plasma cells were identified as events with high CD138 and high CD38 expression density. Clusters of normal and abnormal plasma cells were defined using a 2D projection of all 9 fluorescent parameters (radar plot). An abnormal phenotype was defined as plasma cells with diminished expression of CD19, CD27, CD38, CD45, and/or CD81; overexpression of CD56; and asynchronous expression of CD117 and CD200. **(B)** Comparison of staining without and with different drop-in antibodies (represents example of drop-in verification).

The aim of the drop-in verification was to compare the accuracy of the assessment of minimal residual disease in patients with plasma cell myeloma while using additional antibodies. Five apheresis products and three bone marrow samples were stained in Duraclone RE PC tube parallel without additional anitibody, with CD3 and CD117 ECD drop-in. The addition of drop-in antibody did not compromise the performance of Duraclone RE PC tube staining (see Fig. 1B). Final frequencies of malignant plasma cells between different stainings of the same sample ranged from 0% to 0.01% of CD45+ cells whereas normal plasma cell frequencies ranged from 0% to 0.07% of CD45+ cells.

Furthermore, CD3 drop-in antibody was used to extend the analysis of the immune compartment. The frequencies of residual T, B, and NK cells analyzed in 5 leukapheresis products are shown in Table 4.

**Table 4:** Results from five leukapheresis products showing frequencies of malignant and normal plasma cells as well as residual T, B, and NK cells.

Normal Plasma Cells (%CD45+)	Abnormal Plasma Cells (%CD45)	Number of normal plasma cells	Number of abnormal plasma cells	No CD45+ lymphocytes	T cells (% lymph)	B cells (% lymph)	NK cells (% lymph)
0.13%	0.01%	169	2	23205	78.6%	1.25%	9.36%
0.03%	0.04%	680	53	130277	69.78%	0.34%	18.79%
0%	0.01%	6	45	556943	81.32%	0.07%	6.34%
0.04%	0%	1071	1	94378	85.87%	3.82%	8.75%
0.45%	0.02%	9892	76	317374	72.59%	1.21%	23%

### Inter-assay analysis: Precision/Linearity

**Figure 2:**
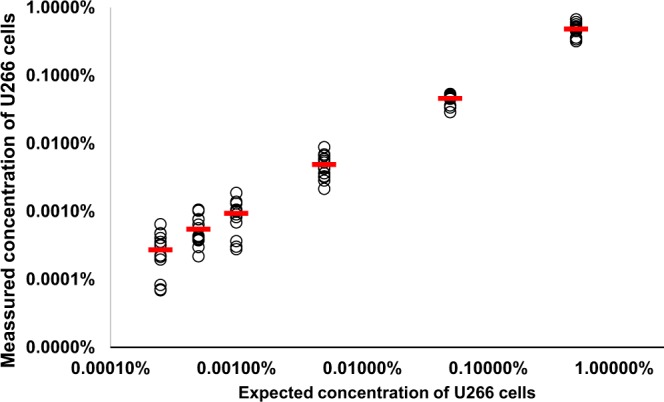
Linearity assessment. Black outlined circles represent data points from 6 different concentrations of U266 cells, spiked into whole blood samples from 5 different normal donors and run in triplicate. Red bars represent the mean of measured values at each concentration. Linear regression delivered a slope of 0.96 and a correlation coefficient of R^2^ = 0.948.

The aim of the first set of experiments of the inter-assay analysis was to evaluate the precision and linearity of assessment of minimal residual disease in patients with plasma cell myeloma. 90 samples with 6 different concentrations of U266 cells across 5 different whole blood samples from normal donors were assayed recording 1,157 × 10^3^ ± 176 × 10^3^ CD45+ events. Samples were created by spiking whole blood from a healthy donor with U266 cells to create samples with known frequencies of this cell line. Final target frequencies ranged from 0.5% to 0.0005% of CD45+ anomalous plasma cells, representing 1 anomalous plasma cell in 2 × 10^3^ CD45+ to 2 anomalous plasma cells in 2 × 10^5^ CD45+ cells, respectively (Fig. 1) as approximated through linear regression by a linear equation y = 0.963 ×−1 × 10^−4^ with a correlation coefficient of R^2^ = 0.948 (Fig. 2). Table 5 shows the results of normal samples spiked with U266 cells. CVs were higher than expected (Poisson noise) for samples with frequencies from 0.5% to 0.005%. Recovery of spiked U266 cells was found at an average of 95% when referring to samples above the theoretical lower limit of detection (LLOD) or quantitation (LLOQ) from 0.5% to 0.005%. LLOD and LLOQ were calculated as described above.

**Table 5:** Results from validation runs on inter-assay linearity/precision (**represents spiked values below the theoretical LLOD).

Spiked %CD45	Recovered %CD45 (mean from 3 replicates in 5 normal whole blood samples)	Relative recovery of target frequency	Number of CD45+ (×10^3^, mean from 3 replicates)	Number of recovered events (mean from 3 replicates)	CV	mean^−1/2^ (“poisson noise”)	theoretical LLOD (20 cells min)	theoretical LLOQ (50 cells min)
0.5000%	0.4815%	96%	1,174	5996	20%	1%	0.0017%	0.0043%
0.0500%	0.0459%	92%	1,153	523	17%	4%	0.0017%	0.0043%
0.0050%	0.0049%	98%	1,157	56	38%	13%	0.0017%	0.0043%
0.0010%**	0.0009%	94%	1,131	10	47%	31%	0.0018%	0.0044%
0.0005%**	0.0005%	109%	1,155	6	47%	40%	0.0017%	0.0043%
0.0003%**	0.0005%	195%	1,164	3	64%	56%	0.0017%	0.0043%

### Inter-assay analysis: Repeatability

In this set of experiments, the aim was to evaluate the repeatability of the assessment. 30 samples from three different sources were assayed: peripheral blood, bone marrow and apheresis product from three different plasma cell myeloma patients. 10 staining replicates from each sample (peripheral blood, bone marrow and apheresis product) were performed and acquired.

382,6 × 10^3^ ± 4,7 × 10^3^ CD45+ events were recorded from 10 replicates of peripheral blood samples. Final frequencies of malignant plasma cells ranged from 0.03% to 0.02% of CD45+, representing 2 anomalous plasma cells in 1 × 10^4^ CD45+ at the CV of 13%. Similarly, 10 replicates of bone marrow samples recorded 355,8 × 10^3^ ± 12,3 × 10^3^ CD45+ events. The estimated frequencies of malignant plasma cells ranged from 0.04% to 0.03% of CD45+ anomalous plasma cells. The %CV was 3. Finally, 10 replicates of autologous apheresis product were analyzed showing 396,4 × 10^3^ ± 3,3 × 10^3^ events. The ranged of from 0.08% to 0.07% of CD45+ anomalous plasma cells. The %CV was 7.

### Inter-operator analysis

**Figure 3:**
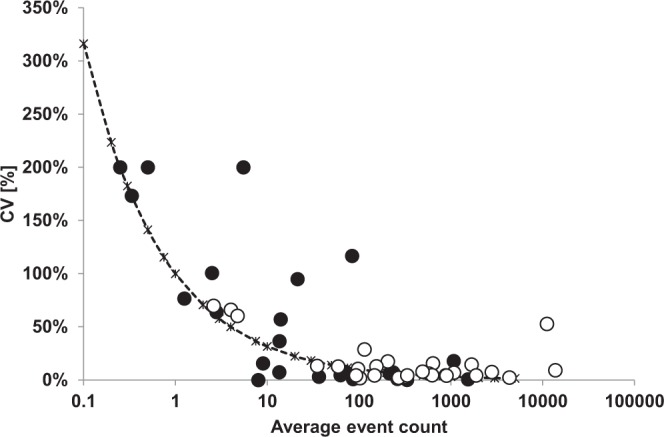
Results from validation runs on inter-operator analysis. White outlined circles represent numbers of normal plasma cells; black circles represent abnormal plasma cells estimated by the analysis.

To evaluate the inter-operator variability a comparison of results from five different data analyses conducted by five independent operators was performed. Trained operators analyzed 25 datasets of apheresis and whole blood samples. The validation data set included 22 unique files and 3 file doublets. Analyses of data sets with average event counts above 50 plasma cell events (malignant or normal), showed high consistency, with CVs lower than 25% (Fig. 3). Outliers of inter-operator CVs were observed in samples with low frequencies ranging from 200% CV for 25 plasma cells total to 0% for 8 plasma cells total.

### Age-of-stain analysis

**Figure 4:**
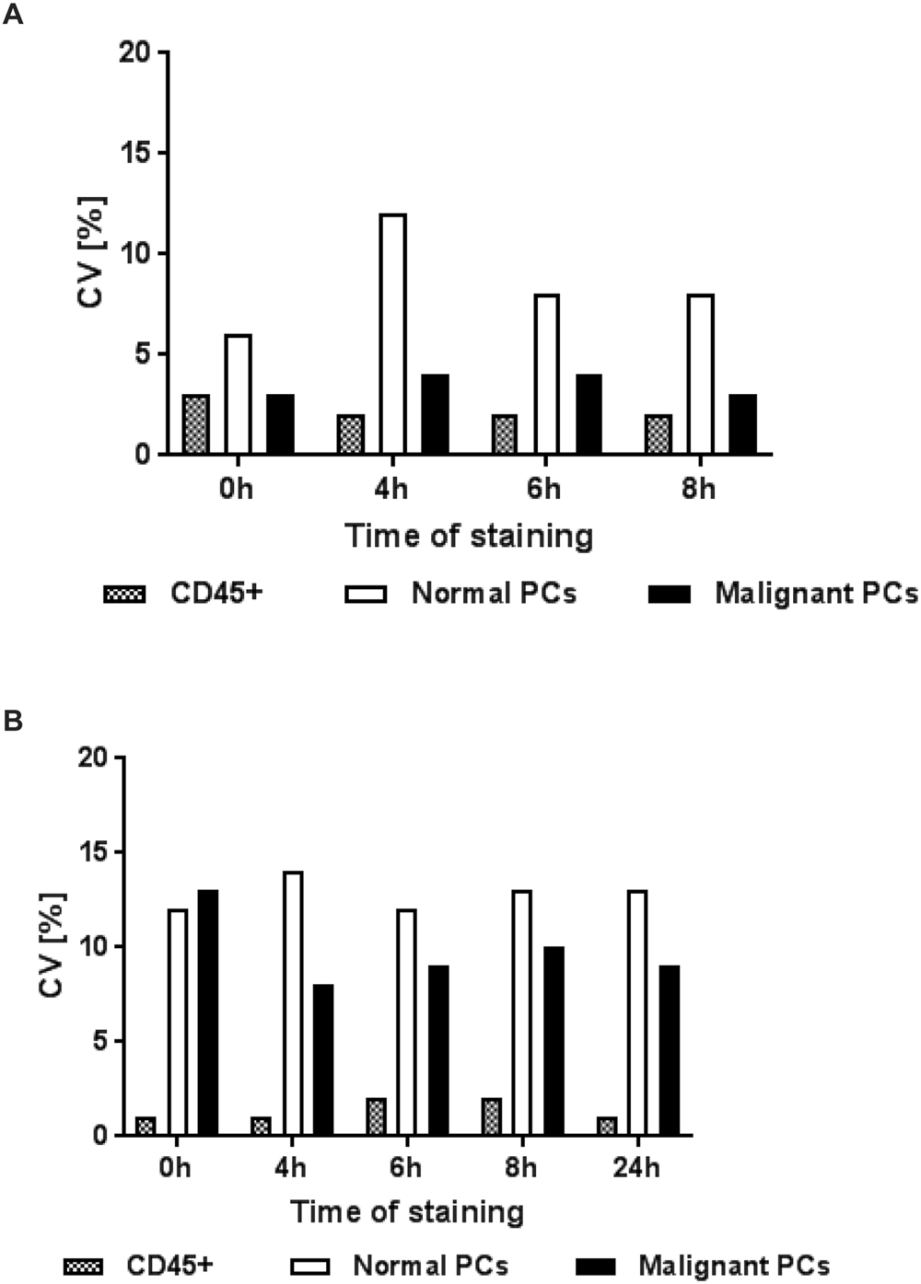
(**A)** Validation run on age-of-stain comparing analysis of 10 replicates from a single bone marrow sample of a myeloma patient at 0, 4, 6 and 8 h. Each column represents a different cell subtype (black/white pattern – CD45+ cells, white – cells with normal plasma cell phenotype, black– cells with malignant plasma cell phenotype) and corresponding CV at different time points of analysis after staining. **(B)** Validation run on age-of-stain comparing analysis of 10 replicates from a single peripheral blood sample of a myeloma patient at 0, 4, 6, 8 and 24 h. Each column represents a different cell subtype (black/white pattern – CD45+ cells, white – cells with normal plasma cell phenotype, black– cells with malignant plasma cell phenotype) and corresponding CV at different time points of analysis after staining.

Age of stain analysis was performed in order to evaluate the stability of the staining over time. 10 replicates from a single bone marrow sample of a myeloma patient were analyzed immediately after staining as well as 4, 6 and 8 h later. The variability for a change from the baseline assessed directly after performed staining ranged from 2 to 13% across different time points. Table 6 and Fig. 4 show the bone marrow age-of-stain analysis with comparable cell frequencies and CVs across all time points.

**Table 6:** Validation run on age-of-stain comparing analysis of 10 replicates from a single bone marrow sample of a myeloma patient at four different time points (0; 4; 6; 8 h).

Age of stain	0 h		4 h		6 h		8 h	
	Mean	SD	Mean	SD	Mean	SD	Mean	SD
CD45+ (×10^3^)	355,8	12,9	396,0	9,5	411,4	8,6	419,8	7,9
Normal plasma cells	210	14	212	27	222	18	203	16
Abnormal plasma cells (×10^3^)	12,1	0,4	12,2	0,5	12,3	0,5	12,2	0,4

Although the reproducibility of the analysis was shown across all sample types (bone marrow, peripheral blood and apheresis (data not shown)) even after 24 h, we note that a moderate shift in granularity, as shown by altered SSC, was observed that required adaptation of the gating.

### Background from blank

In order to determine the limit of blank (LOB) defined as the highest measurement result that is likely to be observed for a blank sample, 25 samples from healthy donors were collected and stained according to standard protocols. The number of detected plasma cells with abnormal phenotype ranged between 0 and 2, while the mean number of CD45+ cells was 1472,5 × 10^3^ and the mean normal plasma cell number recorded was 330. The analysis was performed with a standard protocol and LOB was calculated as the mean result and the standard deviation (SD), following the formula: mean_blank_ +1.645(SD_blank_). The calculated LOB was 0,76.

## Discussion

Flow cytometry is a reliable, easy and cost effective tool for the assessment of minimal residual disease in patients with plasma cell myeloma^26^. Novel drugs significantly improve patients’ outcomes by achieving longer remissions that cannot be measured reliably with standard methods such as immunofixation and electrophoresis^5^. On the other hand, molecular tests like allele-specific oligonucleotide (ASO)-PCR^27^ or next generation sequencing (NGS) of immunoglobulin rearranged genes^28,29^ require pre-treatment evaluation, have relatively high cost per sample and are time consuming. Although flow cytometry MRD testing is being performed by many laboratories, there are major differences in antibody panels, gating strategies and minimal event counts^30^. The effort of minimalizing the laborious workflow and cost while preserving the robustness and sensitivity, has been recently shown by the EuroFlow Consortium^31^ and MSKCC group^32^ (see Table 2).

The presented, standardized assay addresses the urgent need for easy, applicable, lean and sensitive flow cytometry based method for the evaluation of treatment efficacy that can then be routinely used in the clinical setting. Furthermore, this validation evaluated the capability of using different starting material like mobilized peripheral blood and stem cell apheresis for MRD evaluation.

The tested flow cytometry panel was designed based on current recommendation and results of clinical trials^1,2,10^ and took advantage of room temperature stable dried antibodies, preformatted for one test in a assay tube as well as automated instrument setup and compensation routine^33^. Furthermore, analysis using a predefined template, including dynamic gates and radar plots^34^, allowed for a high level of standardization independent of the personnel performing the analysis. This leads to a simplified and easy protocol that can be expanded by including further markers (e.g. ECD and APC-AF700 conjugated fluorochromes), as shown here with the addition of the CD117 or CD3 antibody. Additional antibodies used as drop-ins did not compromise the performance of the assay.

Reliable assessment of minimal residual disease can only be achieved upon stringent validation of the diagnostic assay. Here, we presented a validation study that follows the current recommendation summarized in the consensus guidelines^12,21,23^ with the aim to demonstrate the ability of this assay to detect, in a reproducible and reliable manner, the presence of minimal residual disease.

Validation performed in compliance with ISO15189: 2012 accreditation guidelines for clinical laboratories requires a verification of the accuracy. To validate the accuracy of an assay it is necessary to compare average values obtained with a conventional true value. This presents a technical challenge, since there are currently no fully characterized reference materials available. Furthermore, there are no external quality assessment programs, while other techniques for MRD assessment like (ASO)-PCR or NGS have different sensitivities and specificity, making it difficult to use them as a comparison method^35^. In order to overcome those difficulties and determine the lower level of detection (LLOD), we used whole blood from a healthy donor and spiked it with apheresis product from a patient with plasma cell myeloma with active disease as estimated by standard means. Due to the amount and nature of the original sample the percentage of malignant plasma cells in the samples varied between 0.008% and 0.0005% for CD45+ anomalous plasma cells. CVs were in the expected range: 7–25% (Poisson noise) for all samples. Calculated LLOQ lied at 0.002% and LLOD was shown for 0.001%. In cases of rare populations where the LLOD is 0.01% or lower a CV < 30% is acceptable^22^.

Inter-assay experiments demonstrated linearity of the assay with a linear regression slope of 0.96 and a correlation coefficient of R^2^ = 0.948. Precision of the assay was analyzed in the dilution experiment with samples created by spiking whole blood from a healthy donor with U266 cells to create samples with known frequencies of this cell line. The CVs were higher than expected (Poisson noise) for samples with frequencies from 0.5% to 0.005%; this might be attributed to the modified gating strategy needed for identification of the U266 cellular phenotype as compared to native malignant plasma cells. Analysis of the blank samples from healthy donors showed a low LOB of 0.76. Furthermore, repeatability showed high consistency of the results, regardless of the material used and the age-of-stain for no more than 24 h.

The development and introduction of a highly standardized analysis procedure as well as the introduction of radar plots significantly simplified the analysis procedure. The performed inter-operator analysis showed that inter-operator variability which is highly dependent on the subset abundance^33^ can be significantly reduced. According to our analysis, the consistency in data evaluation with average event counts above 50 plasma cell events (abnormal or normal) can be very high with CVs lower than 25%^11^.

Although this standardized test has been validated to detect at least 20 abnormal plasma cells and quantify the cell concentration above 50 plasma cells, the actual sensitivity will depend on the adherence to the protocol and the quality of the sample^11,24,25^. Since the age of the sample significantly influences the variability, especially in low-abundant cell populations^36^, our assay was performed immediately after sample collection. Age-of-stain analysis showed that immediately following staining, samples could be measured up to 24 h post staining. Variability remained comparable for 24 h.

Our validation results show that a reliable detection of residual myeloma cells was feasible to a detection level of 0.0010% (10^−5^) with a single-tube assay for a variety of materials (peripheral blood, bone marrow and stem cell apheresis).

Our MRD detection approach is applicable to more than 98% of plasma cell myeloma patient samples run in our laboratory. Furthermore, valid estimation of the presence of minimal residual disease was possible from peripheral blood and stem cell products. Due to the high sensitivity and robustness of the assay there was no need of assessment of pretreatment samples from the patients^37^.

In summary, the presented validation results demonstrate a highly standardized, well streamlined and lean workflow approach for the assessment of minimal residual disease in plasma cell myeloma patients. The presented validation of the test followed the FDA-NCI roundtable guidelines^7^ and international consensus recommendations for myeloma flow cytometry based MRD quality control^12,23^.

Expanding the sample material to include peripheral blood and apheresis product will help to collect long-term data of their predictive value as well as extend their usage. Furthermore, wider usage of this MRD assessment standardized approach will allow reliable comparison between laboratories setting new standards in routine evaluation patients’ response to treatment.

## Data Availability

The datasets generated during and analysed during the current study are available from the corresponding author on reasonable request.
